# Picroside III: novel multifunctional iridoid glycoside with promising antileukemic activity

**DOI:** 10.17179/excli2026-9527

**Published:** 2026-06-12

**Authors:** Chang Ha Park

**Affiliations:** 1 Department of Smart Farm, Namseoul University, 91 Daehak-ro, Seonghwan-eup, Seobuk-gu, Cheonan-si, Chungcheongnam-do 31020, Republic of Korea

## ⁯

Picroside III is a compound of considerable interest owing to its distinctive structural features and potential biological activities. However, it remains relatively poorly investigated compared to other members of the picroside family. A PubMed search (2000-2026) revealed that only a limited number of studies have explored its biological functions, whereas Picroside I and II have been extensively studied (Almeleebia et al., 2022[1]). This highlights the need for further investigation of Picroside III.

Picroside III is based on an iridoid glycoside scaffold characterized by a fused cyclopentane and pyran ring system with attached sugar moieties and various functional groups (Guo et al., 2025[2]). Although this core structure is conserved among Picrosides I, II, and III, there are notable differences in the type, position, and stereochemical configuration of substituents.

A key distinguishing feature of Picroside III is its diverse substitution patterns. They are composed of specific aromatic or non-aromatic functional groups attached at distinct positions, which influence the overall electronic distribution and three-dimensional conformation of the molecule (Supplementary information, Figure 1). These structural variations may lead to significant alterations in chemical reactivity and biological activity (Almeleebia et al., 2022[1]). In addition, Picroside III retains a glycosidic linkage, with a sugar moiety attached to a defined position (Supplementary information, Figure 1). Glycosylation enhances aqueous solubility and facilitates molecular transport within biological systems (Hu et al., 2024[3]). Furthermore, the glycosidic bond is susceptible to enzymatic hydrolysis, suggesting that Picroside III may undergo metabolic transformation into various derivatives *in vivo* (Guo et al., 2025[2]).

The distribution of hydroxyl groups is an important structural characteristic of Picroside III. The presence of multiple hydroxyl (-OH) groups enables the formation of hydrogen bonds that can strengthen interactions with enzymes and other proteins (Huan et al., 2023[4]). This capacity for hydrogen-bonding is likely to play a critical role in modulating its biological activity (Yunta, 2017[11]).

Picroside III contains multiple chiral centers, and its stereochemical configuration is largely similar to those of Picrosides I and II, with subtle but important differences. These stereochemical variations influence the manner in which a molecule interacts with specific biomolecular targets, ultimately contributing to differences in biological activity (Huynh et al., 2026[6]). Picroside III exhibits high hydrophilicity, similar to Picrosides I and II. It is readily soluble in water and other polar solvents, which facilitates its mobility in biological systems (Kwon et al., 2025[7]). However, structural differences may result in slight variations in solubility and stability. Pharmacokinetic studies have demonstrated that Picrosides I, II, and III exhibit rapid systemic elimination and extensive tissue distribution following intravenous administration. All three compounds showed a large volume of distribution, indicating efficient penetration into peripheral tissues. Notably, Picroside II exhibited the highest accumulation in the liver, suggesting strong hepatic targeting; Picrosides I and II can cross the blood-brain barrier, unlike Picroside III. These differences in pharmacokinetic behavior are closely associated with structural and physicochemical properties, including hydrophilicity, glycosylation, and substitution patterns, which influence membrane permeability and tissue affinity (Ren et al., 2026[8]). 

Picroside III can participate in various reactions, including hydrolysis, redox processes, and enzyme-mediated transformations, owing to the presence of ester or glycosidic linkages that make the molecule susceptible to enzymatic cleavage *in vivo* (Supplementary information, Figure 1). The resulting metabolites may exhibit biological activities that are distinct from those of the parent compound.

A PubMed search revealed that only four studies reported the biological activities of Picroside III from 2000 to 2026, highlighting its relatively unexplored nature. Picroside III is an iridoid glycoside with a fused cyclopentane-pyran scaffold containing various substituents, hydroxyl groups, and a glycosidic linkage that influences its chemical reactivity, physicochemical properties, and biological activities. Pharmacokinetic studies have demonstrated rapid systemic elimination and extensive tissue distribution, with differences in blood-brain barrier permeability and hepatic accumulation compared with picrosides I and II. The presence of ester and glycosidic linkages render Picroside III susceptible to enzymatic hydrolysis and other transformations, thereby producing metabolites that may exhibit distinct biological effects.

Picroside III exhibits multifunctional biological activities including anti-inflammatory, intestinal barrier-protective, antiviral, and gut microbiota-modulating effects. Importantly, a recent study revealed its antileukemic activity in acute myeloid leukemia (AML) cells, where it promotes differentiation, inhibits proliferation, and induces cell death, suggesting that Picroside III is a promising candidate for further development as a novel anticancer agent. Table 1(Tab. 1) (References in Table 1: Huang et al., 2024[5]; Scott et al., 2022[9]; Sun et al., 2023[10]; Zhu et al., 2015[12]) summarizes the key pharmacological activities reported in previous studies.

## Declaration

### Acknowledgments

Funding for this paper was provided by Namseoul University year 2026.

### Conflict of interest

The author declares no conflict of interest.

### Artificial Intelligence (AI) - assisted technology

The author used an LLM exclusively for improving English language clarity and readability in the manuscript preparation.

## Supplementary Material

Supplementary information

## Figures and Tables

**Table 1:**
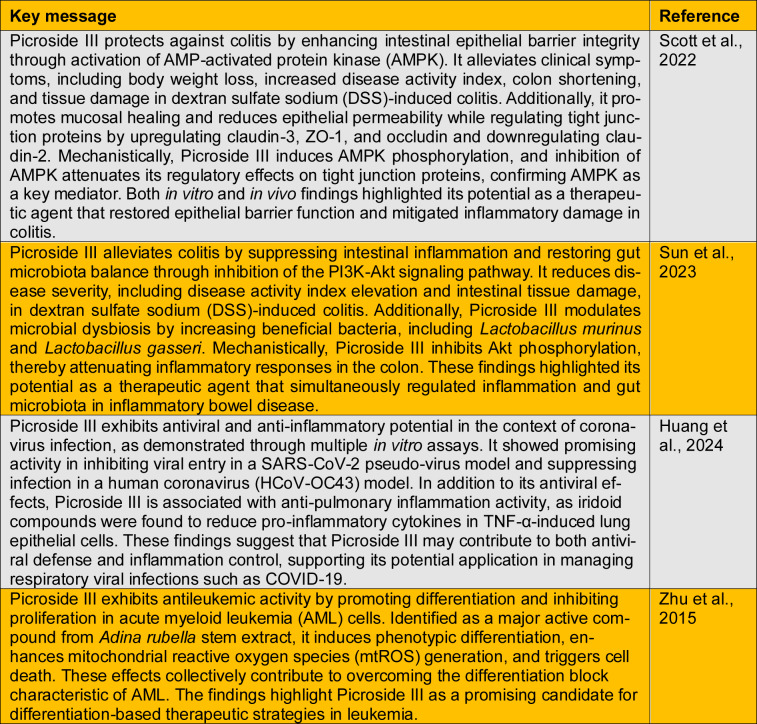
The biological and pharmacological activities of Picroside III according to previous studies
