# Supplementary material for: Picroside III: novel multifunctional iridoid glycoside with promising antileukemic activity
Source: EXCLI J. 2026 Jun 12;25:799–802. doi: 10.17179/excli2026-9527 (PMC13312794; doi:10.17179/excli2026-9527)
Supplement: Supplementary information [file EXCLI-25-799-s-001.pdf]

EXCLI Journal 2026;25:799-802 – ISSN 1611-2156
Received: April 30, 2026, accepted: May 22, 2026, published: June 12, 2026

Supplementary information to

Letter to the editor:

PICROSIDE III: NOVEL MULTIFUNCTIONAL IRIDOID GLYCOSIDE WITH PROMISING ANTILEUKEMIC ACTIVITY

Chang Ha Park[1,*]

[1] Department of Smart Farm, Namseoul University, 91 Daehak-ro, Seonghwan-eup, Seobuk-gu, Cheonan-si, Chungcheongnam-do 31020, Republic of Korea

* **Corresponding author:** Chang Ha Park, Department of Smart Farm, Namseoul University, 91 Daehak-ro, Seonghwan-eup, Seobuk-gu, Cheonan-si, Chungcheongnam-do 31020, Republic of Korea, Tel.: +82-41-580-3254, E-mail: parkch@nsu.ac.kr

https://dx.doi.org/10.17179/excli2026-9527

This is an Open Access article distributed under the terms of the Creative Commons Attribution License (https://creativecommons.org/licenses/by/4.0/).

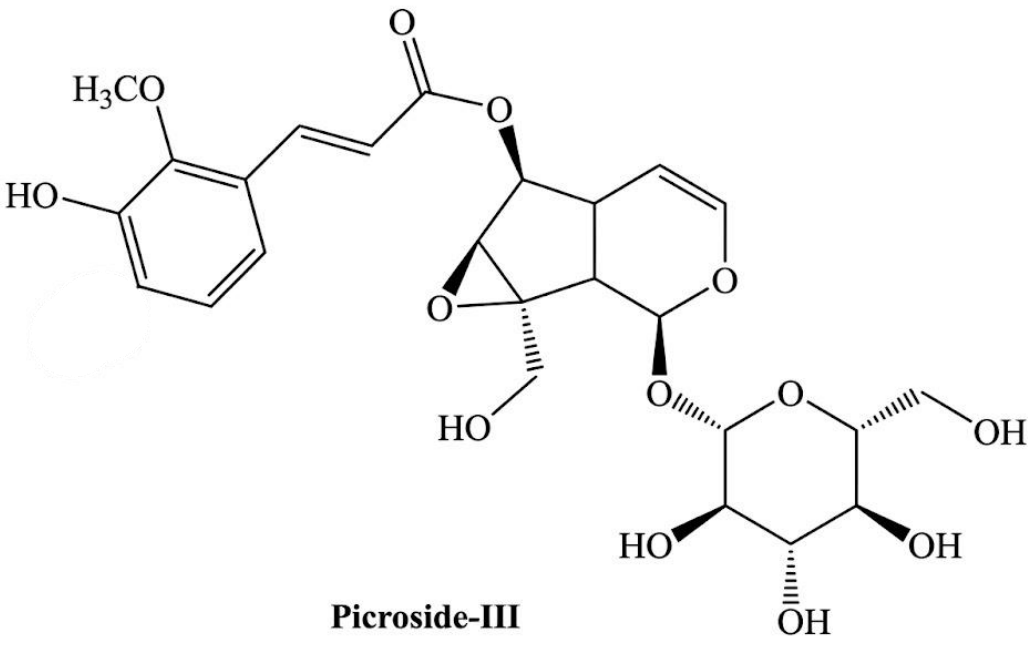

Supplementary Figure 1: Chemical structure of Picroside III
